# Context-specific synthetic T cell promoters from assembled transcriptional elements

**DOI:** 10.21203/rs.3.rs-3339290/v1

**Published:** 2023-10-17

**Authors:** Jacob Appelbaum, Jia Wei, Rithun Mukherjee, Taylor Ishida, James Rosser, Chris Saxby, John Chase, Marc Carlson, Cassie Sather, Wolfgang Rahfeldt, Michael Meechan, Michael Baldwin, Lindsay Flint, Cailyn Spurrell, Joshua Gustafson, Adam Johnson, Michael Jensen

**Affiliations:** Fred Hutch Cancer Center; Seattle Children’s Research Institute; Seattle Children’s Research Institute; Seattle Children’s Research Institute; Seattle Children’s Research Institute; Seattle Children’s Research Institute; Seattle Children’s Research Institute; Seattle Children’s Research Institute; Fred Hutchinson Cancer Research Center; Seattle Children’s Research Institute; Seattle Children’s Research Institute; Seattle Children’s Research Institute; Seattle Children’s Research Institute; Seattle Children’s Research Institute; Seattle Children’s Research Institute; Seattle Children’s Research Institute; Seattle Children’s Research Institute

## Abstract

Genetic engineering of human lymphocytes for therapeutic applications is constrained by a lack of transgene transcriptional control, resulting in a compromised therapeutic index. Incomplete understanding of transcriptional logic limits the rational design of contextually responsive genetic modules^1^. Here, we juxtaposed rationally curated transcriptional response element (TRE) oligonucleotides by random concatemerization to generate a library from which we selected context-specific inducible synthetic promoters (iSynPros). Through functional selection, we screened an iSynPro library for “IF-THEN” logic-gated transcriptional responses in human CD8^+^ T cells expressing a 4–1BB second generation chimeric antigen receptor (CAR). iSynPros exhibiting stringent off-states in quiescent T cells and CAR activation-dependent transcriptional responsiveness were cloned and subjected to TRE composition and pattern analysis, as well as performance in regulating candidate antitumor potency enhancement modules. These data reveal synthetic TRE grammar can mediate logic-gated transgene transcription in human T cells that, when applied to CAR T cell engineering, enhance potency and improve therapeutic indices.

## INTRODUCTION

Current engineered T cell therapies display suboptimal anti-tumor potency and/or excessive toxicities limiting clinical utility^2^. Exogenous transgenes can enhance the anti-tumor immunobiology of immune effector cells, but without adequate regulation pose significant safety risks^3–5^. CARs trigger T cell effector function in response to tumor-associated proteins that would not normally activate native T cells^6,7^. Thus, CAR receptors transform T cells into powerful therapeutic agents, particularly for treating hematological malignancies^8–11^, however, within solid tumors an immunosuppressive milieu can drive CAR T cells toward activation-induced cell death, anergy, or exhaustion ^12,13^. These hypofunctional T cell states limit anti-tumor activity and pose a major hurdle in the application of CAR therapy to solid tumors^14,15^. While CARs generate strong synthetic inputs, the addition of genetic sequences that modulate natural T cell response programs for enhanced functionality, i.e. synthetic outputs, have been more challenging to engineer.

Constitutive expression of recombinant genes in therapeutic T cells can improve anti-tumor activity but may drive adverse side-effects^16–18^, forcing a trade-off between safety and potency enhancement. A regulatory mechanism that activates potency enhancement only in the tumor would offer a layer of safety. For example, SynNotch architecture drives transgene expression in response to ligand engagement by engineered receptors^19,20^. However, SynNotch must be adapted to different target ligands and requires a large DNA footprint to encode necessary components. A more streamlined approach would negate the need for a separate receptor and instead drive expression as a direct transcriptional response to CAR engagement.

A CAR-responsive synthetic promoter could serve this purpose by harnessing the natural signal transduction of T cell antigen stimulation to drive supplemental transgene expression. The simplest synthetic promoters contain repeated endogenous transcription factor response elements (TREs)^19–24^ to amplify signals from endogenous signal transduction pathways. For example, promoters composed of repeat Nuclear factor of activated T-cells (NFAT) TREs have been used to drive exogenous cytokine secretion upon TCR or CAR activation but suffer from leakiness and limited transcriptional activity in the ON-state^22,25,26^. Screens of repeated promoter elements have identified constitutive and inducible promoters in cell lines^27,28^ and stem cells^24^. More complex synthetic promoters could be constructed by combining varied TREs upstream of a minimal (core) promoter^29^, better mimicking natural promoters that utilize combinations of TREs to regulate gene expression by integrating activity of multiple signaling pathways. While these studies interrogated modestly sized libraries created from pre-defined TREs combinations, we reasoned an agnostic search of synthetic promoters formed by combinatorial ligation of selected TREs may efficiently reveal candidates more precisely tuned to defined cellular contexts.

Here, we screen a library of synthetic promoters constructed by a random ligation of T cell activation-specific TREs for candidates that exhibit stringent OFF-state ON-state switching. By encoding the library within a lentiviral vector (LVV) pool and conducting a screen in the context of CAR T cell tumor challenges, we identify transiently active promoters in therapeutically relevant contexts. A supporting computational framework allowed us to identify clusters of promoter behavior in response to antigen stimulation, revealing TRE combinations associated with specific gene expression patterns. This approach selected inducible synthetic promoters (iSynPros) that exhibit stringent OFF states and strong induction following *in vitro* antigen stimulation. iSynPro induction was specific to antigen stimulation, as exposure to co-stimulation alone, Toll like receptor agonists, or stimulatory cytokines failed to activate transcription. When integrated into CAR T cells delivered *in vivo* to tumor bearing mice, the selected synthetic promoter iSynPro1 showed robust inducibility that was spatially and temporally restricted to antigen presence. iSynPro1-driven expression of potency-enhancing synthetic transgenes enhanced CAR T cell function and prevented off target toxicity *in vivo*. This paradigm of rational formulation and random assembly leverages existing clinical CAR designs to enhance T cell potency through regulated expression of supplemental transgenes and has the potential to be applicable to other emerging cellular therapies.

## RESULTS

### Generation of an inducible synthetic promoter library.

**Figure 1:**
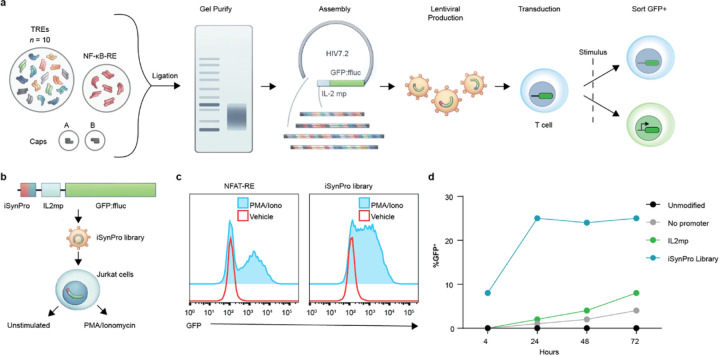
Random ligation of transcriptional response elements establishes an inducible synthetic promoter library. **a,** Schematic of synthetic promoter library assembly and introduction to T cells to screen candidate inducible promoters. **b,** Schema to assess inducibility of iSynPro library in Jurkat cells. **c,** Flow cytometry of GFP:ffluc expression under NFAT or synthetic promoter library regulation in unstimulated and PMA/Ionomycin-treated Jurkat cells. **d,** Flow cytometry time course for GFP expression in Jurkat cells by either no promoter, an IL-2 minimal promoter (IL2mp) or the iSynPro library.

We posited that random ligation of rationally selected TREs would generate a library of cell state responsive synthetic promoters. This library could then be screened for prescribed transcriptional outputs in a cellular context of choice – here we explore “IF-THEN” transcription in antigen-activated CAR T cells (Fig. 1a). We first identified genes upregulated in CD8^+^ T cells within 12 to 48 hours of T cell activation (drawn from clusters I, II, III and VI in Best et al. 2013). Next, transcription factor (TF) binding sites within the promoters of representative genes were identified by cross-referencing the TRED^30^ and TRANSFAC^31^ databases, with eleven TF binding sites appearing in both. We interrogated the JASPAR database^32^ to identify TRE sequences associated with each of the eleven TFs to serve as functional units of our promoter library. Promoters were assembled by random ligation of pooled double-stranded oligonucleotides encoding TREs. Ligation reactions included a 3x amount of the nuclear factor-κB (NF-κB) TRE and a 1x amount of all other TREs, as CARs bearing a 4–1BB domain are known to engage NF-κB^33,34^. Following gel electrophoresis (Extended Data Fig. 1a), DNA products between approximately 200 and 500 base pairs were recovered and cloned into a LVV transfer plasmid upstream of an IL-2 minimal promoter (IL2mp) and a green fluorescent protein:firefly luciferase fusion (GFP:ffluc). The resulting plasmid library was packaged into LVV, yielding a functional titer of 1.16 x 10^9^ infectious units per mL, corresponding to 2.2 x 10^8^ total infectious units.

We retained DNA samples from the TRE ligation product (“*raw*”), the cloned plasmid library (“*plasmid*”) and cDNA from the LVV (“*virus*”) for evaluation by next generation sequencing (NGS). The variable length templates and absence of an alignment reference present a challenge for NGS analysis, which is exacerbated by the block structure of sequences constructed from a limited set of TREs. Accordingly, we devised a computational approach for recovery and analysis of promoter sequences (Extended Data Fig. 1b,c). A first module, *alignMerge*, reconstructs the underlying sequence templates by first sliding and concurrently aligning each read against its pair. A second module, *TREsCaller*, annotates each reconstructed synthetic promoter with TREs from our starting set while accounting for sequencing noise. We used the resulting promoter sequences, rendered as strings of TREs (i.e. TRE contigs) for downstream analysis.

The raw library diversity, as assessed using Lorenz distributions, proved highly polyclonal with a vast majority of promoter sequences occurring only once (Extended Data Fig. 1d). This likely reflects the large potential diversity of synthetic promoters created by our method, of which we were only able to sample a small proportion. The plasmid library showed a skewing in clonality, possibly due to differential clonal expansion after transformation into bacteria. This skewing was retained in the lentiviral library, as demonstrated by strongly correlated TRE contig frequencies among the lentiviral and plasmid libraries (Extended Data Fig. 1e). The lentiviral library showed enrichment of shorter promoters (as measured in TRE units), compared to the plasmid library (Extended Data Fig. 1e,f), likely reflecting a lentiviral packaging bias toward shorter plasmid sequences.

To assess the gene regulatory potential of TRE-based synthetic promoters in bulk, we stimulated human Jurkat T cells with PMA and ionomycin following transduction with our lentiviral promoter library driving a GFP reporter (Fig. 1b). In comparison to Jurkat T cells transduced with a GFP reporter driven by repeated NFAT responsive elements (NFAT-RE), TRE-based synthetic promoters achieved similar levels of induced GFP expression at 24 hours (Fig. 1c). A time course showed GFP expression as early as four hours post-stimulation (Fig. 1d). We observed re-induced GFP expression upon a second stimulation demonstrating some members of the library achieved repeat inducibility (Extended Data Fig. 2a-c). This response demonstrates our library contains inducible synthetic promoters (iSynPros).

### Selection of antigen-responsive iSynPro clones in CD19(4–1BB)CAR T cells

**Figure 2:**
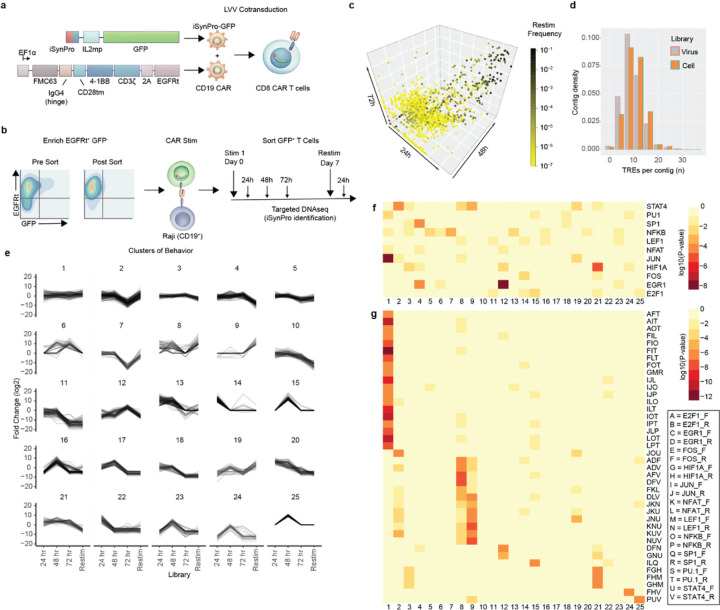
iSynPros respond to CAR stimulation with variant expression patterns associated with combinations of TREs. **a,** Schematic depicting the co-transduction of the iSynPro library and CD19CAR LVVs into CD8^+^ T cells. **b,** Experimental approach to enrich CAR^+^ GFP^-^ cells, stimulate with CD19^+^ LCL tumor cells, and collect GFP^+^ populations for NGS. **c,** Multi-dimensional representation of promoter frequency distributions across the co-culture experiment. 24-, 48- and 72-hour time points are represented along the x, y and z axes, respectively. Promoter frequency observed after restimulation is indicated by color gradient. **d,** Density histogram of contigs comparing promoter lengths (measured in TRE units) between virus and cell libraries. **e,** Clustering of synthetic promoters based on patterns of observed frequencies across co-culture timepoints measured as fold-change (log_2_) from corresponding baseline levels in virus library. **f,** Heatmap of enriched individual TRE frequencies among clusters. Numbers on x-axis indicate cluster groups from **e.** Color gradient indicates statistical significance determined by Fisher’s Test. **g,** Heatmap of enriched three-TRE motifs statistically significant in at least one cluster. Numbers on x-axis indicate cluster groups from **e.** Color gradient indicates statistical significance (by Fisher’s Test). TREs are encoded by single letters, as shown in the key in the lower right.

To select the antigen responsive iSynPros within the iSynPro library, we co-transduced CD8^+^ T cells with the iSynPro LVV library and a separate LVV encoding a CD19-targeting CAR^9^ (Fig. 2a). We first sorted the resulting T cell population for CAR^+^ GFP^-^ T cells, thereby excluding promoter sequences with constitutive expression. To identify synthetic promoter sequences that exhibit increased transcription following antigen exposure, the sorted CAR^+^ GFP^-^ population was co-cultured with CD19^+^ tumor cells for CAR-specific stimulation. GFP^+^ T cells were recovered by flow sorting 24, 48, and 72 hours after an initial tumor challenge and 24 hours after a second tumor challenge one week later. We isolated DNA from recovered T cells and performed targeted NGS to identify inducible promoter sequences as described previously (Fig. 2b).

We first investigated whether the frequency of individual TREs within the promoter library changed during the time-course. We found that, compared to the proportion of TREs in the reference libraries (raw, plasmid, virus), individual TRE proportions among tumor activated GFP+ CD19CAR T cells exhibited low variation throughout our screen (mean coefficient of variation = 0.108, range 0.021 – 0.305, Extended Data Fig. 3a). We next cataloged the abundance of synthetic promoters among inducible GFP^+^ CAR T cells in response to tumor co-culture across timepoints. In contrast to TRE proportions, which did not change during the course of our screen, we found many promoters decreased in frequency at later time points, while few others increased (Extended Data Fig. 3b). After discarding minimally detected promoters (< 4 counts per million at each time point post tumor co-culture) we projectedpromoter frequencies in multi-dimensional space, linking promoter abundance across collection time points. We found a subset of promoter sequences were dominant throughout, while others were abundant only within one or several time points (Fig. 2c). We also found longer synthetic promoters, harboring more TRE units, were over-represented in the cell populations compared to virus (Fig. 2d). Together these data indicate selective preference for additive or synergistic TRE combinations and suggest that promoter frequency is reflective of promoter expression dynamics.

To further examine the potential role of sequence complexity within individual promoters, we studied changes in frequencies of each TRE contig across all collection points in relation to the lentiviral library (taken as a baseline). Fold-change time course trajectories revealed evidence of clustering, as evaluated by the Hopkins statistic (**H** = 0.91, *p* < 10^-16^)^35–37^. Accordingly, we clustered the dynamic behaviors of individual TRE contigs to reveal broad patterns (Fig. 2e). While some promoter sequences were present at similar frequencies across all timepoints, others exhibited increased or decreased abundance following antigen stimulations.

We next explored whether individual or combinations of TREs were enriched within behavioral clusters. For a handful of clusters, we found enrichment of individual TREs (Fig. 2f), while for others we did not. Some TREs enriched in solo were also enriched as parts of a combinatorial motif. Concurrently, there were other enriched motifs that did not have TRE sub-components enriched individually (Fig. 2g). These findings suggest that TREs, both individually and in combination, form functional units underlying synthetic promoter behavior patterns. Enrichment of complex TRE motifs within clusters indicate synergism of individual TREs reflective of a “TRE grammar”. The kinetic clusters and inherent TRE signatures could serve as a resource to inform design of promoters with behaviors appropriate to specific cell therapy applications.

### Recursive and persistent antigen activates iSynPro1

**Figure 3:**
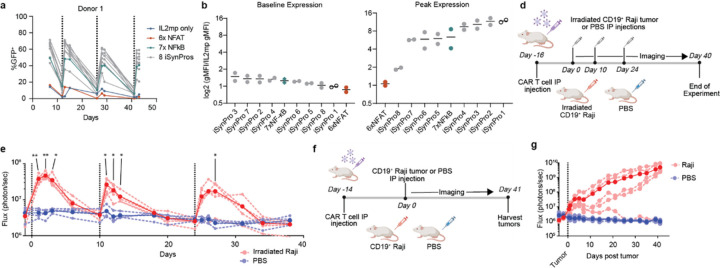
iSynPro1 responds to recursive and persistent antigen stimulation. **a,** Percent GFP induction by a set of synthetic promoters, including eight iSynPro library candidates, in response to recursive antigen stimulation in CD8^+^ CD19CAR T cells. Vertical dashes lines indicate times of stimulation with CD19^+^ LCL cells. One donor is shown here, and an additional donor is shown in Extended Data Fig. 4a. **b,** Baseline GFP expression by synthetic promoters immediately measured prior to each of three stimulations shown in a. Bars show median values (n = 2 donors). **c,** Peak GFP expression of synthetic promoters measured one day after each of three stimulations shown in **a**. **d,** Timeline of the *in vivo* study modeling recursive antigen stimulation. **e,** iSynPro1-driven luciferase activity in response to pulsed treatments of PBS or irradiated CD19^+^ Raji tumor cells following the schema outlined in **d**. Vertical dashed line indicate times of PBS or tumor injections. *n* = 4 mice in PBS treatment group; *n* = 5 mice in Raji treatment group. q values was determine using unpaired multiple T test with false discovery rate set at 1%. **f,** Timeline of the *in vivo* study modeling persistent antigen stimulation. **g,** iSynPro1-driven luciferase activity in response to PBS or CD19^+^ Raji tumor cells. Vertical dashed line indicates PBS or tumor injection. *q* values are denoted: *, *q* < 0.01, **, *q* < 0.001; exact q values available in Supplemental Table 5. Illustrations observed in **d** and **g** were generated using BioRender.

We identified 24 TRE contigs that were (*i*) present at a baseline read frequency in the viral library of at least 0.001 (i.e. 1 in 1000 reads), (*ii*) exhibited a fold change of >1.25 at multiple time points during cell selection and (*iii*) were among the most abundant TRE contigs (comprising 90% of the cell library) (Supplementary Table 2). We the generated lentiviral vectors encoding GFP:ffluc reporters driven by either individual iSynPros, control promoter sequences composed of only NFAT or NF-κB responsive elements, or an “empty” IL2mp-only construct. Finally, manufactured CD8+ CAR T cell products via co-transduction of a second generation CD19CAR lentivirus and iSynPro or control lentiviral vectors and then assessed iSynPro induction potential following antigen exposure. Eight of eight iSynPro sequences tested (numbered in Supplementary Table 2) induced GFP expression upon each recurrent exposure to CD19^+^ LCL and returned to baseline levels after two weeks (Fig. 3a, Extended Data Fig. 4a). Induction was also observed for the NF-κB control promoter, but not for the IL2mp, nor the NFAT control promoters. Because individual iSynPros showed a range of dynamic behaviors, we ranked promoters in order of decreasing baseline expression and increasing peak expression of GFP (Fig. 3b,c). Several iSynPros showed lower background expression and higher peak expression than the NF-κB promoter. Among sequences with the desired features of low background and high induction potential, one sequence, identified as iSynPro1 (Extended Data Fig. 4c), exhibited the most stringent OFF state and greatest degree of transcription induction.

We further investigated the iSynPro1 transcriptional response to both pulsed and persistent *in vivo* antigen exposure. In a first model we adoptively transferred iSynPro1-GFP:ffluc/CD19CAR CD8^+^ T cells into NOD *scid* gamma (NSG) mice, then injected irradiated Raji cells (a CD19^+^ human B lymphoma line) or PBS as a control 16, 26, and 40 days later (Fig. 3d). Following the initial injection of Raji tumor cells, mice harboring iSynPro1-GFP:ffluc CD19CAR T cells showed significant increases in luciferase activity at 24 h (*P* = 0.000599), 48 h (*P* = 0.00011), and 72 h (*P* = 0. 0.003269), compared to mice injected with PBS alone (Fig. 3e). Five days following tumor injection, increases in bioluminescence imaging were no longer significant (*P* = 0.08). Imaging at similar timepoints following subsequent tumor injections revealed continued OFF-ON-OFF luciferase activity patterns and minimal fluctuation in response to PBS (Fig. 3e).

We further assessed the response of iSynPro1 to prolonged antigen exposure *in vivo*, using a model that creates a slowly progressive intraperitoneal lymphoma despite CD19CAR T cell infiltration. In this model, iSynPro1-GFP:ffluc/CD19CAR CD8^+^ T cells are adoptively transferred to NSG mice, followed injection of CD19^+^ Raji tumor cells in numbers sufficient to result in tumor engraftment (Fig. 3f). We tracked iSynPro1 bioluminescence before and after adoptive transfer of Raji tumors and observed increasing iSynPro1 signal over 41 days (Fig. 3g). At the end of the experiment, *ex vivo* flow analysis of tumor biopsies from mice showed sustained CD19 antigen presence (Extended Data Fig. 4d). Interestingly, one Raji-injected mouse showed attenuated iSynPro1 activity. Necropsy at end of study revealed that this mouse was tumor-free (data not shown), consistent with a model in which the CD19CAR T cells eliminated the tumor and subsequently iSynPro signal returned to its OFF state. In summary, persistent antigen exposure led to iSynPro1 activation over a prolonged time course. Our findings with both transient and persistent tumor exposure affirms that iSynPro1 responds to antigen-mediated CAR and TCR signaling.

### iSynPro1 induction is restricted to signal 1 T cell activation pathways

**Figure 4:**
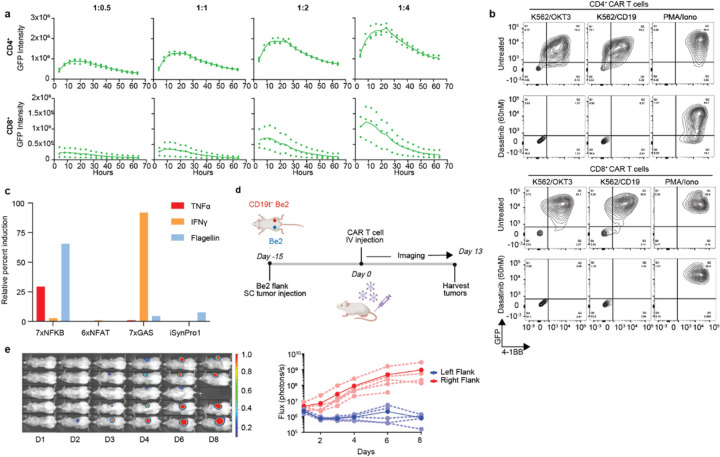
iSynPro1 induction is restricted to signal 1 T cell activation pathways. **a,** Incucyte monitoring of GFP expression in CD19CAR and iSynPro1-GFP:ffluc/CD19CAR T cells in response to co-culture with CD19^+^ Be2 neuroblastoma cells at four effector to target ratios (*n* = 3 donors). **b,** Flow cytometric scatterplots showing induction of GFP and expression of endogenous 4–1BB in iSynPro1-GFP:ffluc/CD19CAR T cells when treated with stimulatory ligands and chemicals with or without the addition of 60 nM dasatinib. Representative donor shown; additional donors in Extended Data Fig. 5b. **c,** GFP expression in Jurkat lines driven by iSynPro1 or control promoters (7x NF-κB, 6x NFAT, 7x GAS) after stimulation with TNFα, IFNγ or Flagellin. Induction percentages are relative to observed induction levels after stimulation with PMA/iono by cell line. **d,** Timeline of the *in vivo* study assessing anatomical localization of iSynPro1 activation. **e,** iSynPro1-driven luciferase activity in CD19^-^ vs. CD19^+^ flank tumors. Bioluminescent imaging (left) and activity quantification in treated mice (right). Illustrations observed in **d** were generated using BioRender.

Antigen encounters promote immunoreceptor tyrosine activation motif (ITAM) phosphorylation transmitting a T cell activating stimulus known as “signal 1”^38^. We investigated whether signal 1 was responsible for iSynPro1 transcriptional induction. We therefore correlated mRNA and protein abundance in CAR T cells harboring iSynPro1-driven GFP:ffluc following antigen stimulation. To ensure equal gene dosage of CD19CAR and iSynPro-GFP:ffluc modules, we utilized transposase-based integration of a single dual-promoter construct housing iSynPro1-regulated GFP:ffluc and a constitutively expressed CD19CAR (iSynPro1-GFP:ffluc/CD19CAR). Live cell fluorescent imaging revealed iSynPro1 GFP induction occurred within 6 hours of stimulation, and transcriptional output positively correlated with antigen abundance (Fig. 4a). We also observed iSynPro1 induced expression of the GFP:ffluc transgene, following overnight CD3/CD28 stimulation by flow cytometry (Extended Data Fig. 5a). Because accumulation of stable GFP proteins can mask rapid transcriptional off rates, we assessed transcript levels by reverse transcriptase digital droplet PCR. We found induction of iSynPro1 within one day post stimulation, with a decrease in expression over the next 8 days while transcript levels of the constitutively expressed CD19CAR did not fluctuate (Extended Data Fig. 5b). Therefore, iSynPro driven transcription induction is rapid and limited to periods of several days following stimulation.

Antigen stimulation of T cells triggers a set of signal transduction/gene expression pathways leading to T cell activation and effector function, some of which overlap with signaling pathways activated by receptors of the innate immune system. To explore the specific signaling inputs necessary or sufficient for iSynPro1 activation, we exposed iSynPro1-GFP:ffluc/CD19CAR T cells to a set of stimulatory ligands and chemicals. We found T cells receiving a stimulus through CD3ζ (via TCR-CD3 complex or CAR stimulation) resulted in iSynPro1 induction, and that this activation could be abrogated by addition of dasatinib, an inhibitor that blocks activity of SRC family kinases involved in proximal TCR/CAR signaling (including LCK)^39,40^ (Fig. 4b, Extended Data Fig. 5c). T cells activated with PMA and ionomycin (PMA/Iono), which bypasses the membrane-proximal signaling cascade of TCR engagement, elicited iSynPro1 induction resistant to dasatinib inhibition. Co-culture of CD19CAR T cells with CD19^-^ tumor cells bearing CD80 and CD86 alone had no effect on iSynPro1-driven transgene expression, demonstrating that co-stimulation via CD28 is not sufficient for iSynPro1 induction (Extended Data Fig. 5d). Likewise, compared to 7X NF-κB and 7X gamma-interferon activation site (GAS) promoters, iSynPro1 does not respond to stimulation with TLR5 agonist flagellin, IFN𝛾, nor TNF. Because iSynPro1 activation is less responsive to these stimulatory molecules, it offers the benefit of restricting gene expression to areas and times of antigen exposure (Fig. 4c). These results show LCK-dependent pathways activated TCR/CAR signaling via CD3ζ are sufficient for iSynPro1 induction, while costimulatory signals and inflammatory cytokines are not.

While our studies thus far have featured the CD19CAR, we hypothesized that other CARs featuring the CD3ζ domain would likewise lead to iSynPro1 induction. Coculture of antigen expressing tumor cells with two additional CAR specificities currently under clinical investigation (B7H3-^41^ and EGFR-targeted CAR T cells^42^) also resulted in iSynPro1 activation (Extended Data Fig. 5e). Thus, we find that activation of iSynPro1 is both CAR specificity-agnostic and likewise tumor-agnostic, as iSynPro1 induction in CD19CAR T cells occurs upon exposure to a variety of human tumor cell lines transfected to express CD19 (Extended Data Fig. 5f). These data are consistent with a model in which iSynPro1 responds via signal 1.

The restriction of iSynPro1 transcriptional activation to antigen-stimulated T cells may also allow spatial regulation of gene expression *in vivo*. To test the localization of iSynPro1 activation, we assessed iSynPro1 activity in in NSG mice bearing subcutaneous SK-N-Be(2) tumors (human neuroblastoma, hereafter referred to as Be2) modified to express CD19 (Fig. 4d). We administered CD8^+^ iSynPro1-GFP:ffluc/CD19CAR T cells intravenously fifteen days after injection of control CD19^-^ Be2 tumors into the left flank and CD19^+^ Be2 tumors into the right flank of NSG mice. To evaluate the spatial activation of iSynPro, bioluminescent imaging was conducted every four to seven days. We observed luminescence (Fig. 4e) and GFP expression (Extended Data Fig. 5g) within the right flank CD19^+^ tumor, but not the left CD19^-^ flank tumor, demonstrating the iSynPro1-driven gene expression is restricted to anatomical areas with antigen presence.

### An iSynPro1-driven transgene improves CAR T cell function.

**Figure 5:**
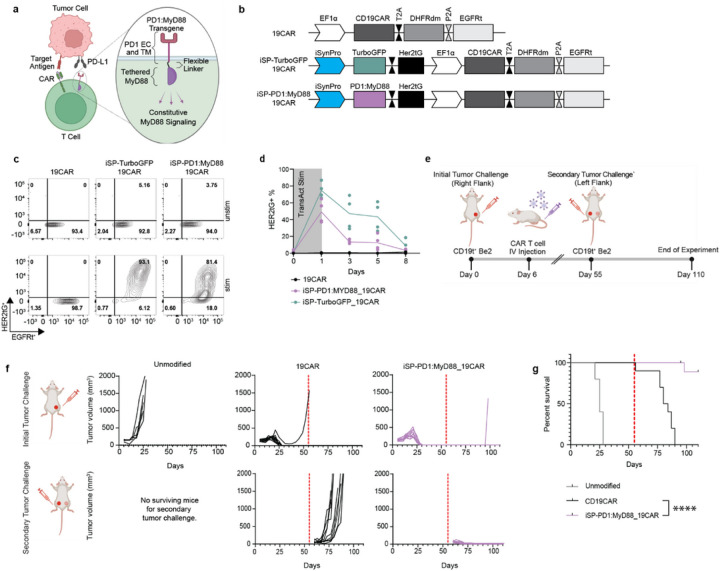
iSynPro1-regulated expression of a potency-enhancing transgene improves CAR T cell functionality. **a,** Schematic of the PD1:MyD88 potency enhancing transgene. **b,** Schematic of the *piggyBac* transposon-based vectors used to introduce CD19CAR alone or alongside iSynPro1(iSP)-driven TurboGFP or PD1:MyD88 into primary human T cells. **c,** Flow cytometric contour plots of a representative donor showing surface marker expression of EF1- and iSP-driven cassettes in unstimulated or CD19^+^ Raji stimulated CD8^+^ CD19CAR T cells modified with constructs described in **b.** **d,** Transgene expression time-course by flow cytometric quantification in iSP1-PD1:MyD88/CD19CAR and iSP1-TurboGFP/CD19CAR CD8^+^ T cells stimulated with TransAct for 16 hours. Lines indicate mean. (*n* = 4 donors). **e,** Timeline of the *in vivo* study assessing CAR T cell ability to control CD19^+^ Be2 tumor growth upon recursive subcutaneous tumor injections. **f,** Tumor volume measurements after initial and secondary challenges with CD19^+^ Be2 tumor cells. Vertical dashed lines indicate the time of secondary tumor challenge. **g,** Kaplan Meier survival curve comparing Marker-Only and PD1:MyD88 groups. P value was determined by Mantel-Cox test; (n = 10 mice per group). The vertical dashed line indicates the time of secondary tumor challenge. *p* values are denoted: ****, *P* < 0.0001. Exact *P values available in* Supplemental Table 5 Illustrations observed in **a** and **e** were generated using BioRender.

Within the field of CAR T cell therapy, there is an unmet need to develop T cell products with potency enhancements. Potency enhancement of CAR T cell products could be mediated by promoters featuring tumor-responsive activity so that effects of transgenes are swiftly deployed upon tumor encounter and extinguished after tumor clearance. We designed a PD1:MyD88 transgene with dual functionality to abrogate CAR T cell deactivation by PD1 ligands and supplement CAR T cell activation via MyD88 signaling^43,44^ (Fig. 5a). We then assessed whether this novel immune switch receptor would be an effective potency enhancement^45,46^ when regulated by logic-gated iSynPro1 transcription.

We first evaluated transcriptional profiles of CD19 CART cell products harboring either iSynPro1-PD1:MyD88 or iSynPro1-GFP by performing RNAseq on sorted CD4+ and CD8+ T cell products prior to and following an *in vitro* tumor challenge (Extended Data Fig. 6 a-d). Among differentially expressed genes we detected high induction of the PD1:MyD88 or GFP transgenes (as expected). Within iSynPro1-PD1:MyD88 containing CAR T cells, we also detected upregulated expression of IFNG, IL1B, IL2 and CSF2, suggesting enhanced effector function and supporting the immunomodulatory role of iSynPro1-PD1:MyD88. We therefore used PD1:MyD88 as a model payload to evaluate potency enhancement by a deployed iSynPro1 regulated transcription module.

We designed a set of dual-promoter *piggyBac* transposon-based vectors incorporating CD19CAR together with iSynPro1-driven PD1:MyD88 or TurboGFP (Fig. 5b). Our vector design included the 2A separated marker tags appended to CD19 CAR (EGFRt) and PD1:MyD88 (HER2tG) to track constitutive and iSynPro-driven expression via flow cytometry. Following *in vitro* manufacture, T cell products demonstrated constitutive expression of the CAR cassette and activation-dependent induction of the iSynPro1 module, as measured by HER2tG (Fig. 5c, Extended Data Fig. 7 a,b). We reasoned that because MyD88 signaling activates NF-κB motifs, which are present in iSynPro1, a potential self-sustaining positive feedback loop may emerge. We therefore reassessed the stringency of iSynPro1 regulation following introduction of the PD1:MyD88 transgene. Following a CD3/CD28 stimulation, we found that iSynPro1 ON-OFF state transitions were similar when driving expression of PD1:MyD88 or a control, TurboGFP, indicating that iSynPro1 maintains stringent expression control in the context of expressing PD1:MyD88 (Fig. 5d).

To understand the potency enhancing effects of iSynPro1 regulated PD1:MyD88, we subjected T cells to a series of *in vitro* tumor challenges every three days. We utilized live cell fluorescent imaging to monitor CAR T cell cytotoxicity and proliferation in response to challenges with CD19t^+^/mCherry^+^ Be2 tumor cells (Extended Data Fig. 7c). Tumor tracking revealed that the addition of iSynPro1 regulated PD1:MyD88 expression resulted in marked reduction in tumor signal 3 days after the third tumor challenge whereas control CD19CAR T cells lost capacity to inhibit tumor outgrowth (Extended Data Fig. 7d). Simultaneously, iSynPro1-regulated PD1:MyD88 CAR T cells exhibited increased survival and proliferation in response to repeated tumor exposures (Extended Data Fig. 7e).

We further evaluated the impact of iSynPro1-regulated expression of PD1:MyD88 in an *in vivo* tumor rechallenge model in which CD19CAR T cells were challenged two successive exposures to subcutaneous CD19^+^ Be2 tumors (Fig. 5e). Following engraftment of the initial tumor, mice subsequently treated with iSynPro1-PD1:MyD88 CD19CAR T cells controlled both initial and secondary tumor challenges (Fig. 5f), resulting in markedly improved survival (Fig. 5g). These findings provide confirmation that iSynPro1-regulated PD1:MyD88 is a CAR T cell potency enhancing genetic module. These results also provide a basis for the design of potency-enhanced CAR T cell products wherein the synthetic module is deployed selectively by CAR T cells that have trafficked to tumor and engaged target antigen.

### iSynPro1 regulation of a mitogenic potency enhancement prevents T cell overgrowth.

**Figure 6:**
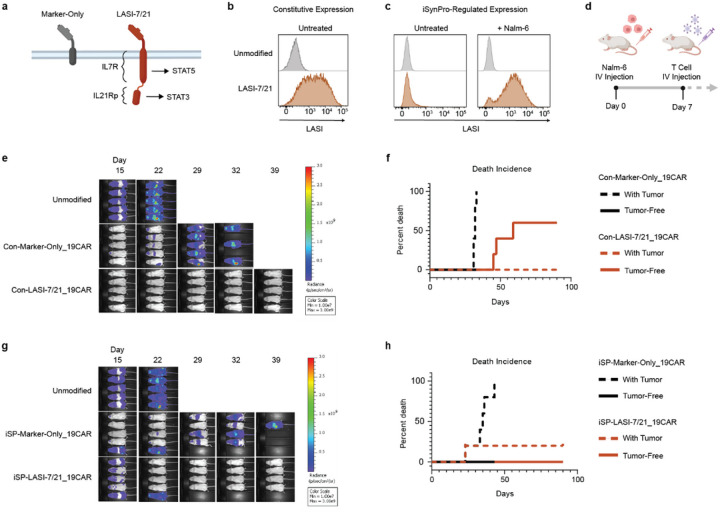
iSynPro1 regulation retains potency enhancement of a mitogenic transgene *in vivo* and prevents subsequent T cell overgrowth. **a,** Schematic of LASI-7/21 protein, composed of IL7 and IL21R domains, and Marker-Only control. **b,** Flow cytometric analysis of LASI-7/21 expression in primary human T cells under regulation by a constitutive promoter. **c,** Flow cytometric analysis of LASI-7/21 expression in primary human T cells under regulation by iSynPro1, untreated or co-cultured with CD19^+^ Nalm-6 tumor cells. **d,** Experimental outline for *in vivo* assessment of CD19CAR T cells bearing constitutively expressed or iSynPro1-regulated LASI-7/21. **e,** Bioluminescent images of mice bearing Nalm-6 tumors expressing firefly luciferase. Mice were treated with unmodified or CD19CAR T cells with constitutively expressed supplemental transgenes. **f,** Death incidence in tumor-bearing and tumor-free mice treated with CD19CAR T cells with constitutively expressed supplemental transgenes. **g,** Bioluminescent images of mice bearing Nalm-6 tumors expressing firefly luciferase. Mice were treated with unmodified or CD19CAR T cells with iSynPro1-regulated supplemental transgenes. **h,** Death incidence in tumor-bearing and tumor-free mice treated with CD19CAR T cells with iSynPro1-regulated supplemental transgenes. Illustration in part **d** was generated using BioRender.

CAR T cell potency enhancement via transgenic pro-growth and pro-survival signals increases the risk of T cell mediated toxicity. Our group has developed a ligand-autonomous STAT inducer protein that provides constitutive IL-7 and IL-21 signals (LASI-7/21), including activation of STAT5 and STAT3 (Fig. 6a). LASI-7/21 incorporates a constitutively active IL7R mutant originating in T cell leukemia^47^ and an appended IL21R peptide. Transposon mediated delivery of the LASI-7/21 transgene into primary human T cells under control of constitutive and iSynPro promoters (Extended Data Fig. 8a) achieved constitutive (Fig. 6b) and antigen-dependent expression of LASI-7/21 (Fig. 6c).

Continuous STAT5 and STAT3 signaling delivered by LASI-7/21 may contribute to CAR T cell growth^48^ and survival^49^, respectively. To assess this possibility, we examined the effects of CD19CAR T cells bearing constitutively expressed LASI-7/21 against the human leukemia cell line Nalm-6 in NSG mice (Fig. 6d). Constitutive expression of LASI-7/21 led to improved tumor clearance (Fig. 6e), however 60% of mice became moribund after tumor clearance (Fig. 6f), suggesting **LASI-7/21 caused T cell overgrowth and subsequent toxicity**. A CAR T cell lymphoproliferative disorder driven by LASI-7/21 is further suggested by necropsy findings that included enlarged and discolored livers and spleens (Extended Data Fig. 8b).

We hypothesized that signal 1-dependent expression of LASI-7/21 by iSynPro1 could ensure that T cell growth enhancement is self-limited upon tumor clearance. To test this possibility, we assessed the safety and efficacy of CAR T cells bearing iSynPro1-regulated LASI-7/21 *in vivo* (Extended Data Fig. 8c). Indeed, iSynPro1-regulated LASI-7/21 enhanced CAR T cell tumor clearance, achieving undetectable Nalm-6 levels in four of five mice (Fig. 6g, Extended Data Fig. 8d). Remarkably, despite efficacy improvements by iSynPro1-regulated LASI-7/21, no adverse health events or deaths were observed in mice following tumor clearance (Fig. 6h). This resulted in significantly improved survival compared to mice treated with CAR T cells bearing iSynPro1-regulated marker-only control (Extended Data Fig. 8e). Examination of iSynPro1-regulated LASI-7/21 CAR T cells in the peripheral blood of mice revealed T cell numbers decreased after tumor clearance (Extended Data Fig. 8f). Among circulating human T cells, iSynPro1 limited LASI-7/21 expression to 1.8% of cells at day 35, compared to 74.6% of cells in constitutive LASI-7/21 controls (Extended Data Fig. 8g). Taken together, we conclude that iSynPro1 regulation enables simultaneous CAR T cell functional enhancements by a mitogenic transgene while preventing toxicity due to unrestricted T cell growth.

## DISCUSSION

Endogenous promoters achieve fine-tuned temporal and stimulation-responsive regulation of gene expression via the combinatorial effects of cis- and trans-acting regulatory elements^50,51^. Stoichiometric and spatial relationships of DNA-bound transcription regulators integrate with protein activity states and other factors to specify gene expression levels^52,53^. Currently, incomplete understanding of TRE combinatorial logic precludes rational design of compact promoters responsive to T cell receptor antigen stimuli. Our approach circumvents incomplete knowledge of the complex architecture governing eukaryotic gene expression to permit *de novo* identification of promoter element combinations with desirable regulatory patterns.

Accordingly, we hypothesized libraries of synthetic promoters composed of randomly arrayed regulatory elements could yield synthetic promoters that impart transcriptional regulation specific to contextually defined cellular inputs. We constructed a promoter library through random assembly of T cell associated regulatory elements and tuned a high throughput screen to identify promoters with features attractive for application in CAR T cells including activation state dependence, stringent OFF states, a tuned range of magnitude and kinetics, and recursive state switching. Therefore careful selection of screening parameters ensured the applicability of regulated synthetic promoters to sophisticated CAR T designs.

The findings from our screen can guide future endeavors to build synthetic promoters. We found that concatemerization of curated TREs that bind transcription factors mediating specific cell status modules resulted in a diverse library of sequences that could be selected in a cellular context specific for intended applications. This strategy of rational formulation and random assembly, followed by functional selection in context has potentially broad applications for cell type and context-specific derivation of synthetic promoters that regulate therapeutic transgene expression.

We investigated the mechanism and context dependence of iSynPro1 activation when deployed in CAR T cells. We showed that iSynPro1 induction depends on signal 1 of T cell activation and is unresponsive to other T cell activation inputs. The quiescence of iSynPro1 following addition of ligands that activate NF-κB signaling (despite the presence of NF-κB TREs) demonstrates the specificity of transcriptional regulation achieved by TRE combinations. Further, the requirement for signal 1 to activate iSynPro1 restricts the potential of payload expression due to other environmental stimuli. Indeed, iSynPro1 activity *in vivo* only occurred within antigen-bearing tumors, highlighting the spatial control of transgene expression afforded by this system.

The synthetic promoter described here may also serve as a tool in other contexts requiring the linkage of gene expression to T cell activation. First, iSynPro1 could be used as a reporter for T cell activation, thereby streamlining screening processes for identification of antigen-specific T cell clones or elimination of tonically signaling CAR designs. Second, utilization of iSynPro1 as a gate within a cellular logic circuit could trigger expression of a secondary and more promiscuous CAR, which would otherwise pose the risk of off-tumor toxicity. Third, regulated expression could limit safety risks posed by constitutive production of immunomodulatory cytokines such as IL12, IL18, and IL15, which exhibit promising preclinical T cell anti-tumor anti-tumor potency enhancements^3–5^. Our approach therefore is a general method to equip CAR T cells with regulated expression of elements that augment anti-tumor function.

Subsequent studies may address some of the shortcomings of this approach. Our synthetic promoter library was constructed from 11 T cell activation-specific TREs ligated as contiguous blocks. Increasing library complexity could better mirror naturally occurring promoters, for example by inclusion of variable spacing between TREs, inclusion of a wider set of TREs, or by including TREs from orthogonal gene sets. To overcome the significant clonal skewing we observed during incorporation of our ligated ‘raw’ library into plasmids, repeated ligation/cloning reactions could permit evaluation of additional promoter candidates. Nevertheless, despite the sparse exploration of the large numbers of potential promoters that could result from random ligation, isolation of many suitable iSynPros suggests our library design approach efficiently produced promoter sequences capable of repeated transcriptional induction.

The work presented here demonstrates the utility of synthetic promoters assembled from candidate TREs identified using analysis of gene expression and transcription factor networks. Such a strategy yields a rich library and eliminates the need for exhaustive design-build-test iterations. Moreover, the library when formulated as a lentiviral pool allowed for at-scale selection in primary human T cells. Feature optimization of parameters such as ON state magnitude, OFF state stringency and kinetic induction of thousands of unique clones can be explored for particular use cases. While our work has focused on engineering therapeutic T cells, this strategy should allow discovery of novel regulatory logic operable in a wide variety of potentially therapeutic primary cells, organoids, or iPS-derived cell lines for subsequent clinical deployment.

## METHODS

### TRE sequence motifs

T cells relevant TRE sequence motifs were identified by first searching the TRED^30^ and TRANSFAC^31^ database for T cell-centric transcription factors. Eleven transcription factors were present in both databases. For each transcription factor, we obtained the corresponding TRE sequence from the JASPAR^32^ database of transcription factor binding profiles.

### Synthetic promoter library design

TRE-specific and NruI- or XbaI-containing sense and anti-sense 5’ phosphorylated oligonucleotides were resuspended in 100 µM in STE buffer (100 mM NaCl, 50 mM Tris–HCl, 1 mM EDTA, pH 7.8, Sigma Aldrich). Equal volumes of sense and anti-sense oligos were mixed, incubated at 95 °C for 3 min, and gradually cooled down to room temperature. The synthetic promoter library was constructed by ligating these blocks at appropriate stoichiometric molar ratios (6x of NF-κB TRE block, 2x of each other TRE, 1x NruI block, and 1x XbaI) with high concentration T4 DNA ligase (Thermo Fisher).

Resultant fragments ranging from 50 to 500 bp were gel extracted and ligated into NruI:XbaI digested HIV lentiviral backbone,^54^ upstream of IL2mp and GFP:ffluc. The ligation product was used to transform *dam-/dcm-* competent *E. coli* (NEB). A sample of the transformed cells was transferred to a kanamycin agarose plate and the remaining cells cultured in 50 ml LB + kanamycin (50 µg/mL) medium. DNA was extracted using Qiagen Plasmid Midi kit (Qiagen).

### Construct design

All studies involving lentivirus featured the FMC63 CD19CAR cloned into an HIV backbone^55^. PiggyBac-transposon constructs were designed with a human elongation factor 1 alpha (EF1a) promoter driving expression of five-part polycistron: 1) human G01S anti-CD19CAR^56^, 2) P2A ribosomal skip sequence^57^, 3) double mutant of the dihydrofolate reductase (DHFRdm) for drug selection^58^, 4) T2A ribosomal skip sequence, and 5) a truncated epidermal growth factor receptor (EGFRt) to serve as cell surface marker^59^. iSynPro-regulated transgene sequences, including GFP:ffluc, TurboGFP, PD1:MYD88,; LASI and Marker Only control^55^, were introduced into piggyBac constructs in forward or reverse orientation upstream of the EF1a-driven cassette. All constructs were assembled from synthesized DNA fragments using standard molecular biology techniques.

### Lentivirus production and titering

Lentivirus was produced by co-transfecting the HIV7 transfer plasmid and vectors pCHGP-2, pCMV-Rev2 and pCMV-G encoding packaging proteins (Rev, Gag, Pol, VSVG) in HEK293T cells using Lipofectamine 2000 (Invitrogen). Viral supernatants were harvested 72h after transfection, filtered through a 0.45 µM filter, and centrifuged at 24,500 rpm. The virus pellets were resuspended in serum free medium and stored at −80 °C. Lentiviruses were titrated in Jurkat cells by flow cytometry analysis of the marker expression (e.g. EGFRt, GFP, etc.) and Lenti-X p24 Rapid Titer Kit (Clontech).

### Cell line culture

HEK293T cells were cultured in DMEM (Invitrogen) supplemented with 10% FBS (Hyclone), 2.5% HEPES (Invitrogen), 1% L-Glutamine (Invitrogen), and 1% sodium pyruvate (Invitrogen). SK-N-Be2 (also referred to as Be2) cells were cultured in DMEM supplemented with 10% FBS and 1% L-Glutamine. Jurkat, Tm-LCL, Raji, K562, DHL-4 and SupB15 suspension cells were cultured in RPMI (Invitrogen) supplemented with 10% FBS and 1% L-Glutamine.

### Primary T cell isolation and culture

Human peripheral blood mononuclear cells (PBMCs) derived from blood discard kits of healthy donors (BloodworksNW) were isolated by Ficoll-Paque (Pharmacia Biotech). CD4^+^ and CD8^+^ cells were purified from the PBMC using RoboSep (Stemcell). In some instance, the CD4^-^, CD8^-^ PBMC fraction was retained. CD4^+^ T cells were cultured in RPMI with 10% FBS, 5 ng/ml rhIL-7 (Miltenyi) and 0.5 ng/ml rhIL-15 (Miltenyi). CD8^+^ cells were cultured in RPMI with 10% FBS, 50 U/ml rhIL-2, (Chiron Corporation) and rhIL-15 (0.5 ng/ml). In some instances, CD4^+^ or CD8^+^ cells were cultured in 50 U/mL rhIL-2, 20 ng/mL IL-4 (Milteyni), 10 ng/mL IL-7 (Milteyni) and 20 ng/mL Il-21 (Milteyni). Medium was replaced twice a week.

### T cell transduction and expansion

CD4^+^ or CD8^+^ T cells were resuspended to 2 to 4 x 10^6^ cells / mL in RPMI medium supplemented with cytokines as above and stimulated with Dynabeads™ Human T Activator CD3/CD28 (Invitrogen) at 1:1 ratio overnight. The activated T cells were added with protamine sulfate (40 ug/ml, APP Pharmaceuticals)) and then transferred into a 12-well plate. Viruses were thawed, vortexed and added to each corresponding well. For co-transduction, both viruses were added to the cells at certain ratios. For example, in iSynPro library screening, CD19CAR-T2A-EGFRt virus and the iSynPro-IL2mp-GFP:ffluc library virus were co-transduced at MOI of 2 and 0.1 respectively. The plate was then transferred to 37°C after spinoculation for 30 minutes at 800 x g. Equal volume of warm complete RPMI media supplemented with cytokines was added to each well at 4 hours or overnight post transduction. Six days post transduction, CD3/CD28 beads were removed from the cells and the transduction efficiency was checked by flow cytometry analysis of cell-surface marker or GFP expression.

For T cells transduced with CD19CAR-T2A-EGFRt-T2A-DHFRdm, methotrexate (MTX) was used to select transgene-containing cells. MTX was added to the T cell culture medium at 50 µM 6 days after transduction and supplemented when medium was changed. Transduced T-cells were expanded by stimulation with irradiated (8000 rad) TM-LCL at a 1:7 E:T ratio in the presence of 50U/mL IL-2 (CD8^+^), 5ng/mL IL-7 (CD4^+^) and 1ng/ml IL-15. The *in vitro* and *in vivo* assays were performed 10 days after T cell expansion.

### T cell nucleofection and expansion

CD4^+^ or CD8^+^ cells were nucleofected using 10 nM donor plasmid and 10 nM RNA (encoding for piggyBac transposase) with the Lonza 4D system using pulse program EO-115. Nucleofected CD4^+^ or CD8^+^ cells were transferred to a G-REX culture vessel pre-seeded with a CD4^-^/CD8^-^ PBMC fraction in X-Vivo 15 (Lonza) supplemented with 2% KnockOut serum replacement (ThermoFisher), 80 U/mL IL-2 (StemCell), 20 ng/mL IL-4 (Miltenyi), 10 ng/mL IL-7 (Miltenyi) and 20 ng/mL IL-21 (Miltenyi). Media was replaced twice weekly and cell expanded for 21 days.

### Sorting of CD8/iSynPro-GFP/CAR T cells using FACS

Eight days after CD19CAR and iSynPro-GFP:ffluc co-transduction, CD8^+^ T cells were sorted for EGFRt^+^GFP^-^ population by FACS to exclude cells containing constitutively expressing synthetic promoters.

Cells were resuspended in PBS/10% FBS staining solution at 1 x 10^6^/mL, filtered (30 µm filter) and stained with APC conjugated anti-EGFRt (APC-cetuximab, custom made by BD Biosciences). Cells were washed with the staining solution, resuspended at 1x 10^6^/ml for cell sorting (FACSJazz™, BD Biosciences). The sorted EGFRt^+^GFP^-^ cells were pelleted, pooled and seeded in RPMI supplemented with IL-2/IL-15 and 1X Penicillin /Streptomycin (Gibco).

Four days later, these T cells were washed with RPMI medium to remove the cytokines and co-cultured with irradiated Tm-LCL cells at 1:2 ratio. The cell suspension was harvested separately at 24, 48 and 72h after co-culturing, resuspended in 1X PBS+10%FBS and filtered (30 µm filter). The GFP positive population was sorted out by FACS. The sorted cells were centrifuged down and stored at −80°C until DNA extraction.

### NGS library preparation

Libraries were generated from DNA samples prepared from the plasmid, virus and GFP sorted cell populations (Fig. 1b and 2a) for sequencing on the Illumina Miseq platform as follows. DNA was extracted using QIAamp DNA Micro Kit (Qiagen #56304) and concentration measured by Qubit DNA HS assay (Thermo Fisher Scientific #Q32851). A first round PCR was performed for targeted amplification of the synthetic promoter library (refer sequence schema below). First round PCR primers comprised of (1) a locus-specific region (letters in ‘bold red’) that matched invariant bases immediately adjacent to the 5’ and 3’ ends of the variable synthetic promoter region (letters in italics), and (2) a linker oligo (green overhang) to permit subsequent conjugation of Illumina adapter sequences. For efficient library recovery, a touchdown PCR protocol was employed with KAPA HiFi HotStart Ready Mix (KAPA Biosystems #KK2601). Each reaction used 100 ng of DNA template based on our PCR optimization result. Samples with material in excess of 100 ng were split into several PCR reactions. Resulting products were combined and purified by Agencourt AMPure XP (Beckman Coulter #A63880).


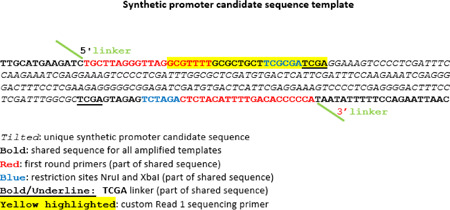


From 15ng of each first round PCR reaction, dual index amplicon libraries were generated by adding Illumina Index-Adapter sequences to the DNA templates. This was accomplished by a second round PCR with KAPA HiFi HotStart Ready Mix (KAPA Biosystems #KK2601) using Illumina Nextera-XT Index primers that incorporate linker oligos at their 3’ end (Illumina #FC-131–1001). The shared linker oligo between primers of both rounds of PCR facilitate conjugation of the Index-Adapters to the DNA templates. Completed libraries were purified with Agencourt AMPure XP beads (Beckman Coulter #A63880) and quantified using Qubit DNA HS assay (Thermo Fisher Scientific #Q32851). Separately, a PCR-Free amplicon library was prepared from 100ng of purified DNA sourced from the raw ligation product with the Accel-NGS 2S DNA Library Kit (IDT #10009877). This PCR-Free library was quantified by qPCR using the KAPA Library Quant Kit (Roche Diagnostics #KK4824). Following each PCR round, amplicon libraries were quality assessed by Agilent’s 2200 TapeStation D5000 assay (Agilent #5067–5588).

### Sequencing

All sequencing was performed on an Illumina MiSeq with MiSeq Reporter v2.5.1 (Illumina), using Illumina’s v3 reagents (#MS-102–3003) and following manufacturer’s instructions. Our libraries featured amplicons with stretches of monotemplate sequence (~30 bases) at both ends juxtaposing variable synthetic promoter sequences in between – this is problematic for Illumina sequencers and can result in poor quality reads. As a diversity countermeasure, 10% PhiX Control v3 Library (Illumina FC-110–3001) was supplemented for sequencing. From this attempt it was evident significantly higher PhiX levels would be needed to improve read quality, in turn sacrificing usable read throughput. This prompted a search for custom Read 1 and 2 primers that would bypass the monotemplate sequence in our libraries. We identified a suitable candidate for use as Read 1 primer (refer previous sequence schema) but were unable to design a custom Read 2 primer counterpart. For Read 2, we relied on the Illumina supplied sequencing primer mix, that primes off the 3’ linker. Illumina sequencer color matrix calibrations that are based on the initial bases of Read 1 are of primary importance – thus we proceeded with the custom Read 1 primer (GCGTTTTGCGCTGCTTCGCGATCGA) and to work in conjunction with it, a custom PhiX Read 1 Primer (ACACTCTTTCCCTACACGACGCTCTTCCGATCT). We spiked 10% PhiX Control to support improved quality for Read 2. Such an approach greatly improved the overall quality of both Read 1 and Read 2. The raw library templates which lacked terminal monotemplate bases representative of plasmid backbone sequences and were directly ligated to Illumina adapters, were accordingly sequenced with the default Illumina sequencing primers. PhiX was added as a quality control for cluster generation, sequencing, alignment and matrix calibration. Sequencing configuration was 300 bp paired end. Read recovery is listed in Supplementary Table 3.

### Transient and repeated stimulation of CD19CAR+ iSynPros-GFP:ffluc T cells

For repeated *in vitro* stimulation, T cells were stimulated with CD3/CD28 Dynabeads overnight and co-transduced with CD19CAR and iSynPros-GFP:ffluc. On day 7, beads were removed, and a sample of the cells was analyzed for EGFRt and GFP expression by flow cytometry. The remainder of the cells were continuously cultured for 7 days, stimulated with irradiated (8000 rad) TM-LCL at 1:2 ratio for 16 to 18 hours and analyzed by flow cytometry. This procedure was repeated two more times on 14-day intervals.

### *In vitro* Incucyte assay

Incucyte (Sartorius) live imaging was used to track the cytotoxicity, proliferation, and iSynPro1 driven GFP expression CAR T cells. CD19t^+^/mCherry^+^ Be2 tumor cells and CAR T cells were co-cultured in triplicates in 384-well imaging plates. The number of tumor cells and CAR T cells that were seeded varied depending on the E:T ratios of interest. For the serial re-challenge experiment, subsequent doses of Be2 tumor cells were added every 3 days for a total 9 days. Images were captured every 4-hours at 10x or 20x magnification. IncuCyte 2020C software was used for analysis. Cytotoxicity of CAR T cells, represented by the target cell presence, was quantified by RCU. GFP expression upon iSynPro1 induction was quantified by GCU. Cytotoxicity and iSynPro1 induction were assessed using the Basic Analyzer analysis. Proliferation of CAR T cells was quantified using Non-Adherent Cell-by-Cell analysis.

### Dasatinib assay

CD4^+^ and CD8^+^ CAR T cells expressing iSynPro1-GFP:ffluc were co-cultured with unmodified, OKT3^+^, or CD19t^+^ K562 target cells at a 1:1 E:T ratio, or stimulated with PMA/Ionomycin, with or without Dasatinib at 60 nM. After 16 hours, cells were harvested and analyzed by flow cytometry.

### Co-stimulation assay

CD4^+^ and CD8^+^ CAR T cells expressing iSynPro1-GFP:ffluc were left untreated or co-cultured with unmodified, CD19t^+^, CD80^+^CD86^+^, or CD19t^+^CD80^+^CD86^+^ K562 target cells at a 1:1 E:T ratio. After 16 hours, cells were harvested and analyzed by flow cytometry.

### Jurkat reporter assay

Jurkat reporter cell lines were generated via lentiviral transduction to contain either 7x NF-κB, 6x NFAT, 7x GAS or iSynPro1 driving GFP expression. Cells were cultured in a base media of RPMI supplemented with 10% FBS and 1% glutamine and cell stimulants, TNFα 50 ng/mL (Bio-Techne), IFNγ 50 ng/mL (Bio-Techne) and PMA/Ionomycin 1x (Thermo Fisher), and TLR5 agonist, flagellin 1.25 ug/mL (InvivoGen) for 16 hours at 37°C. 7xNF-κB served as a positive control for TNFα, and 7xGAS for IFNγ. After the stimulation period, cells were harvested and analyzed by flow cytometry for GFP expression. As the PMA/Ionomycin stimulated condition presented the maximal GFP expression for all Jurkat lines, relative percent induction was calculated by dividing the GFP positive percentage for each stimulation condition by the GFP positive percentage induced by PMA/Ionomycin stimulation in each cell line.

### RNA kinetics study

Cell pellets were collected and spun at 300 X g for 7 minutes, media aspirated and frozen at −80 °C. Pellets were processed for RNA using a RNeasy Mini kit (Qiagen), cDNA was synthesized using a SuperScript™ IV Reverse Transcriptase (ThermoFisher), and cDNA was quantified using the QX-200 droplet digital PCR system (Bio-Rad) according to the manufacturer’s recommendations. Primer and probes sets are detailed in Supplemental Table 5.

### RNA-seq

PD1:MyD88 or GFP expressing CD19CAR CD4^+^ or CD8^+^ T cells were subjected to CD19t and mCherry expressing Be2 cells for 24 hours at a 2:1 effector to target ratio. Following 24h, effectors were subjected to another bolus of Be2 targets and, after an additional 24 hours, effectors and targets removed from culture, labeled with live dead stain and anti-CD4, anti CD8, anti-CD3, Herceptin, and Erbitux antibodies. One million live CD4+ and CD8+ cell populations were sorted using a Sony MA900 cell sorter, and cell pellets were frozen at −80° C in preparation for RNAseq.

Cell pellets were shipped on dry ice to Genewiz for library preparation and sequencing. RNAseq libraries were pooled and sequenced to generate 25M 150bp X 2 reads per sample (Illumina). RNA-seq data was analyzed using the nf-core RNA-seq pipeline version 3.4, code available from nf-core/rnaseq (https://nf-co.re/rnaseq/3.12.0). Briefly, reads were mapped to the human genome (GRCh38) using STAR aligner (v2.6.1d). Gene expression counts were generated using salmon (v1.5.2). Differential gene expression analysis between two phenotypes (iSynPro1-PD1:MYD88 and iSynPro1-GFP) was performed using edgeR version 3.32.1^57^.^57^. First, genes whose expression was less than 10 counts in 2 samples were discarded from further analyses. Across samples, normalization was then performed using trimmed mean of M-values, via the function *calcNormFactors*. Next, *estimateGLMCommonDisp* and *estimateGLMTrendedDisp* were run to properly handle over-dispersion at the global and single gene level. Differentially expressed genes were determined using the glmQLFit function, as those showing a two-sided raw p value <0.01 and |log2(fold change)| >=1.

### *In vivo* studies

All mouse experiments were approved by the SCRI (Seattle Children’s Research Institute) Animal Care and Use Committee. NOD/Scid IL2RγCnull mice were obtained from The Jackson Laboratory or bred in-house.

#### Subcutaneous Single-Challenge Model:

9- to 13-week old NOD/Scid IL2RγCnull (NSG) mice were injected subcutaneously (SC) with 5 X 10^6^ Be2 or Be2/CD19 cells on day 0. Fourteen days later, mice were intravenously (IV) injected with 2.5 x 10^6^ of CD8^+^/iSynPro1-GFP:ffluc/CD19CAR.

#### Subcutaneous Double-Challenge Model:

9- to 13-week old NSG mice were injected SC on the right flank with 5 x 10^6^ Be2/CD19 cells on day 0. Six days later, mice were injected IV with 2.5 x 10^6^ of T cells and monitored thereafter for tumor progression. At any point, mice with tumors that exceeded 1500 mm^3^ were euthanized. On day 55, surviving mice were re-challenged with 5 x 10^6^ Be2/CD19 on the left flank via a SC injection. Mice were thereafter monitored for tumor progression until day 110.

#### Intraperitoneal Raji Model:

10- to 11-week old female NSG mice were injected intraperitoneally (i.p.) with 3 x 10^6^ or 5 x 10^6^ CD8^+^/iSynPro1-GFP:ffluc/CD19CAR cells. 500,000 live or 10 x 10^6^ irradiated Raji (CD19^+^) tumor cells were i.p. injected 15 to 17 days later. The multiple doses of irradiated Raji cells were given through i.p. injections for the rechallenging study.

Bioluminescent imaging was performed weekly by (i.p.) injection of 4.29 mg/mouse D-luciferin (Xenogen), anesthesia by isoflurane and imaging 10 minutes post D-luciferin injection using the IVIS Spectrum Imaging System (Perkin Elmer). Luciferase activity was analyzed using Living Image Software Version 4.3 (Perkin Elmer) and photon flux was analyzed within regions of interest.

#### Systemic Nalm-6 Model:

11–13 week old NSG mice were injected with one million human Nalm-6 leukemia cells via the tail vein, modified to express a fusion protein of mCherry and firefly luciferase. Six days after tumor injection, tumor engraftment in each mouse was quantified via bioluminescent imaging as described above. Mice were then assigned to treatment groups to equalize average tumor engraftment across groups. Seven days after tumor injection, mice were systemically injected via the tail vain with two or four million T cells. Thereafter, mice were monitored for tumor progression by bioluminescent imaging. Mice were euthanized when they showed moderate to severe hind-limb paralysis, an effect of leukemia progression, or otherwise as recommended by veterinary staff. Retro-orbital blood samples were taken on a weekly basis to track T cell engraftment in the peripheral blood.

T Cell Tracking by Retro-Orbital Bleeds. Beginning at day 17 after tumor injection, peripheral blood retro-orbital bleeds of mice were taken on a weekly basis, and flow cytometry was performed on blood samples to quantify T cell engraftment. First, samples were subjected to red blood cell lysis using Pharm Lyse Buffer (BD, Cat. # 555899) and treated with Fc blocking reagent (Miltenyi, Cat. # 130–059-901) to prevent indiscriminate antibody binding. Next, cells were stained with the following panel of reagents: anti-CD3, anti-human CD45, anti-mouse CD45, and fixable viability stain 520 to discriminate live human T cells. Finally, samples were fixed in 0.5% paraformaldehyde (Electron Microscopy Sciences, Cat. # 15713) in PBS before analysis. CountBright absolute counting beads (Invitrogen, Cat. # 2207530) were then added to each sample to allow for calculation of T cell concentrations in analyzed blood.

### Statistical analyses

Data wrangling and visualization were conducted using Prism (GraphPad software), R (www.r-project.com) and RStudio (www.rstudio.com). The number of replicates or T cell donors are indicated in figure legends. Comparisons of means between two groups was conducted using two-tailed t-tests. Enrichment of TREs within promoter clusters was evaluated using one-sided (alternative=”greater") Fisher’s test (see Supplemental Methods for additional details). Survival comparisons following adoptive transfer of tumor xenografts used log-rank (Mantel-Cox) tests. The number of mice are indicated in the figure legends. For all tests, *p* < 0.05 was considered significant and was corrected for multiple comparisons. Exact values of test statistics, degrees of freedom, variance assumptions, and power are listed in Supplementary Table 5.

## Supplementary Material

Supplement 1

## References

[1] ZabidiM. A. & StarkA. Regulatory Enhancer-Core-Promoter Communication via Transcription Factors and Cofactors. Trends Genet 32, 801–814 (2016).2781620910.1016/j.tig.2016.10.003PMC6795546

[2] RafiqS., HackettC. S. & BrentjensR. J. Engineering strategies to overcome the current roadblocks in CAR T cell therapy. Nat Rev Clin Oncol 17, 147–167 (2020).3184846010.1038/s41571-019-0297-yPMC7223338

[3] PegramH. J. IL-12-secreting CD19-targeted cord blood-derived T cells for the immunotherapy of B-cell acute lymphoblastic leukemia. Leukemia 29, 415–422 (2015).2500524310.1038/leu.2014.215PMC5189717

[4] Klebanoff C. A. IL-15 enhances the in vivo antitumor activity of tumor-reactive CD8+ T Cells. Proceedings of the National Academy of Sciences 101, 1969–1974 (2004). 10.1073/pnas.0307298101 PMC357036 14762166

[5] KoneruM., O’CearbhaillR., PendharkarS., SpriggsD. R. & BrentjensR. J. A phase I clinical trial of adoptive T cell therapy using IL-12 secreting MUC-16ecto directed chimeric antigen receptors for recurrent ovarian cancer. J Transl Med 13, 1–11 (2015).2589036110.1186/s12967-015-0460-xPMC4438636

[6] PorterD. L., LevineB. L., KalosM., BaggA. & JuneC. H. Chimeric Antigen Receptor–Modified T Cells in Chronic Lymphoid Leukemia. N Engl J Med 365, 725–733 (2011).2183094010.1056/NEJMoa1103849PMC3387277

[7] EshharZ., WaksT., GrossG. & SchindlerD. G. Specific activation and targeting of cytotoxic lymphocytes through chimeric single chains consisting of antibody-binding domains and the gamma or zeta subunits of the immunoglobulin and T-cell receptors. Proc Natl Acad Sci U S A 90, 720–724 (1993).842171110.1073/pnas.90.2.720PMC45737

[8] LabaniehL., MajznerR. G. & MackallC. L. Programming CAR-T cells to kill cancer. Nat Biomed Eng 2, 377–391 (2018).3101119710.1038/s41551-018-0235-9

[9] GardnerR. A. Intent-to-treat leukemia remission by CD19 CAR T cells of defined formulation and dose in children and young adults. Blood 129, 3322–3331 (2017).2840846210.1182/blood-2017-02-769208PMC5482103

[10] GhorashianS. Enhanced CAR T cell expansion and prolonged persistence in pediatric patients with ALL treated with a low-affinity CD19 CAR. Nat Med 25, 1408–1414 (2019).3147790610.1038/s41591-019-0549-5

[11] MaudeS. L. Chimeric Antigen Receptor T Cells for Sustained Remissions in Leukemia. N Engl J Med 371, 1507–1517 (2014).2531787010.1056/NEJMoa1407222PMC4267531

[12] ThommenD. S. & SchumacherT. N. T Cell Dysfunction in Cancer. Cancer Cell 33, 547–562 (2018).2963494310.1016/j.ccell.2018.03.012PMC7116508

[13] SchietingerA. & GreenbergP. D. Tolerance and exhaustion: defining mechanisms of T cell dysfunction. Trends Immunol 35, 51–60 (2014).2421016310.1016/j.it.2013.10.001PMC3946600

[14] MariathasanS. TGFβ attenuates tumour response to PD-L1 blockade by contributing to exclusion of T cells. Nature 554, 544–548 (2018).2944396010.1038/nature25501PMC6028240

[15] SrivastavaS. Immunogenic Chemotherapy Enhances Recruitment of CAR-T Cells to Lung Tumors and Improves Antitumor Efficacy when Combined with Checkpoint Blockade. Cancer Cell 39, 193–208.e10 (2021).3335745210.1016/j.ccell.2020.11.005PMC7878409

[16] HsuC. Cytokine-independent growth and clonal expansion of a primary human CD8+ T-cell clone following retroviral transduction with the IL-15 gene. Blood 109, 5168–5177 (2007).1735334610.1182/blood-2006-06-029173PMC1890824

[17] HuB. Augmentation of Antitumor Immunity by Human and Mouse CAR T Cells Secreting IL-18. Cell Reports 20, 3025–3033 (2017).2895422110.1016/j.celrep.2017.09.002PMC6002762

[18] MarkleyJ. C. & SadelainM. IL-7 and IL-21 are superior to IL-2 and IL-15 in promoting human T cell–mediated rejection of systemic lymphoma in immunodeficient mice. Blood 115, 3508–3519 (2010).2019019210.1182/blood-2009-09-241398PMC2867264

[19] RoybalK. T. Engineering T Cells with Customized Therapeutic Response Programs Using Synthetic Notch Receptors. Cell 167, 419–432.e16 (2016).2769335310.1016/j.cell.2016.09.011PMC5072533

[20] RoybalK. T. Precision Tumor Recognition by T Cells With Combinatorial Antigen-Sensing Circuits. Cell 164, 770–779 (2016).2683087910.1016/j.cell.2016.01.011PMC4752902

[21] KallunkiT., BarisicM., JäätteläM. & LiuB. How to Choose the Right Inducible Gene Expression System for Mammalian Studies? Cells 8, 796 (2019).3136615310.3390/cells8080796PMC6721553

[22] Hooijberg E., Bakker A. Q., Ruizendaal J. J. & Spits H. NFAT-controlled expression of GFP permits visualization and isolation of antigen-stimulated primary human T cells. Blood 96, 459–466 (2000). 10887106

[23] EdeC., ChenX., LinM.-Y. & ChenY. Y. Quantitative Analyses of Core Promoters Enable Precise Engineering of Regulated Gene Expression in Mammalian Cells. ACS Synth. Biol. 5, 395–404 (2016).2688339710.1021/acssynbio.5b00266PMC4874895

[24] WuM.-R. A high-throughput screening and computation platform for identifying synthetic promoters with enhanced cell-state specificity (SPECS). Nat Commun 10, 2880 (2019).3125379910.1038/s41467-019-10912-8PMC6599391

[25] ChinnasamyD. Local Delivery of lnterleukin-12 Using T Cells Targeting VEGF Receptor-2 Eradicates Multiple Vascularized Tumors in Mice. Clinical Cancer Research 18, 1672–1683 (2012).2229113610.1158/1078-0432.CCR-11-3050PMC6390958

[26] ZhangL. Improving Adoptive T Cell Therapy by Targeting and Controlling IL-12 Expression to the Tumor Environment. Molecular Therapy 19, 751–759 (2011).2128596010.1038/mt.2010.313PMC3070103

[27] BrownA. J., SweeneyB., MainwaringD. O. & JamesD. C. Synthetic promoters for CHO cell engineering. Biotechnology and Bioengineering 111, 1638–1647 (2014).2461526410.1002/bit.25227

[28] Schlabach M. R., Hu J. K., Li M. & Elledge S. J. Synthetic design of strong promoters. Proceedings of the National Academy of Sciences 107, 2538–2543 (2010). 10.1073/pnas.0914803107 PMC2823900 20133776

[29] BlazeckJ. & AlperH. S. Promoter engineering: Recent advances in controlling transcription at the most fundamental level. Biotechnology Journal 8, 46–58 (2013).2289082110.1002/biot.201200120

[30] JiangC., XuanZ., ZhaoF. & ZhangM. Q. TRED: a transcriptional regulatory element database, new entries and other development. Nucleic Acids Res 35, D137–140 (2007).1720215910.1093/nar/gkl1041PMC1899102

[31] MatysV. TRANSFAC and its module TRANSCompel: transcriptional gene regulation in eukaryotes. Nucleic Acids Res 34, D108–110 (2006).1638182510.1093/nar/gkj143PMC1347505

[32] SandelinA., AlkemaW., EngströmP., WassermanW. W. & LenhardB. JASPAR: an open-access database for eukaryotic transcription factor binding profiles. Nucleic Acids Res 32, D91–94 (2004).1468136610.1093/nar/gkh012PMC308747

[33] Li G. 4–1BB enhancement of CAR T function requires NF-κB and TRAFs. JCI Insight 3, (2018). 10.1172/jci.insight.121322 PMC6237232 30232281

[34] PhilipsonB. I. 4–1BB costimulation promotes CAR T cell survival through noncanonical NF-κB signaling. Sci. Signal. 13, eaay8248 (2020).3223496010.1126/scisignal.aay8248PMC7883633

[35] HOPKINS B. & SKELLAM J. G. A New Method for determining the Type of Distribution of Plant Individuals. Annals of Botany 18, 213–227 (1954).

[36] Lawson R. G. & Jurs P. C. New index for clustering tendency and its application to chemical problems. J. Chem. Inf. Comput. Sci. 30, 36–41 (1990).

[37] Wright K. The R Journal: Will the Real Hopkins Statistic Please Stand Up? The R Journal 14, 282–292 (2022).

[38] Bretscher P. A. A two-step, two-signal model for the primary activation of precursor helper T cells. Proceedings of the National Academy of Sciences 96, 185–190 (1999). 10.1073/pnas.96.1.185 PMC15114 9874793

[39] WeberE. W. Pharmacologic control of CAR-T cell function using dasatinib. Blood Adv 3, 711–717 (2019).3081405510.1182/bloodadvances.2018028720PMC6418502

[40] MestermannK. The tyrosine kinase inhibitor dasatinib acts as a pharmacologic on/off switch for CAR T cells. Science Translational Medicine 11, eaau5907 (2019).3127027210.1126/scitranslmed.aau5907PMC7523030

[41] VitanzaN. A. Intraventricular B7-H3 CAR T Cells for Diffuse Intrinsic Pontine Glioma: Preliminary First-in-Human Bioactivity and Safety. Cancer Discovery 13, 114–131 (2023).3625997110.1158/2159-8290.CD-22-0750PMC9827115

[42] RavanpayA. C. EGFR806-CAR T cells selectively target a tumor-restricted EGFR epitope in glioblastoma. Oncotarget 10, 7080–7095 (2019).3190316710.18632/oncotarget.27389PMC6925027

[43] ProsserM. E., BrownC. E., ShamiA. F., FormanS. J. & JensenM. C. Tumor PD-L1 co-stimulates primary human CD8(+) cytotoxic T cells modified to express a PD1:CD28 chimeric receptor. Mol Immunol 51, 263–272 (2012).2250321010.1016/j.molimm.2012.03.023

[44] KaczanowskaS. A Synthetic CD8α:MyD88 Coreceptor Enhances CD8+ T-cell Responses to Weakly Immunogenic and Lowly Expressed Tumor Antigens. Cancer Res 77, 7049–7058 (2017).2905501310.1158/0008-5472.CAN-17-0653PMC5732881

[45] OdaS. K. A CD200R-CD28 fusion protein appropriates an inhibitory signal to enhance T-cell function and therapy of murine leukemia. Blood 130, 2410–2419 (2017).2904236410.1182/blood-2017-04-777052PMC5709784

[46] Oda S. K. A Fas-4–1BB fusion protein converts a death to a pro-survival signal and enhances T cell therapy. Journal of Experimental Medicine 217, (2020). 10.1084/jem.20191166 PMC7953733 32860705

[47] ShumT. Constitutive Signaling from an Engineered IL7 Receptor Promotes Durable Tumor Elimination by Tumor-Redirected T Cells. Cancer Discov 7, 1238–1247 (2017).2883087810.1158/2159-8290.CD-17-0538PMC5669830

[48] BurchillM. A. Distinct effects of STAT5 activation on CD4+ and CD8+ T cell homeostasis: development of CD4+CD25+ regulatory T cells versus CD8+ memory T cells. J Immunol 171, 5853–5864 (2003).1463409510.4049/jimmunol.171.11.5853

[49] OhH.-M. STAT3 protein promotes T-cell survival and inhibits interleukin-2 production through up-regulation of Class O Forkhead transcription factors. J Biol Chem 286, 30888–30897 (2011).2173006910.1074/jbc.M111.253500PMC3162449

[50] OrlandoG. Promoter capture Hi-C-based identification of recurrent noncoding mutations in colorectal cancer. Nat Genet 50, 1375–1380 (2018).3022464310.1038/s41588-018-0211-zPMC6380472

[51] CarninciP. The transcriptional landscape of the mammalian genome. Science 309, 1559–1563 (2005).1614107210.1126/science.1112014

[52] FurlongE. E. M. & LevineM. Developmental enhancers and chromosome topology. Science 361, 1341–1345 (2018).3026249610.1126/science.aau0320PMC6986801

[53] ViselA., RubinE. M. & PennacchioL. A. Genomic views of distant-acting enhancers. Nature 461, 199–205 (2009).1974170010.1038/nature08451PMC2923221

[54] AusubelL. J. Production of CGMP-Grade Lentiviral Vectors. Bioprocess Int 10, 32–43 (2012).22707919PMC3374843

[55] JohnsonA. J. Rationally Designed Transgene-Encoded Cell-Surface Polypeptide Tag for Multiplexed Programming of CAR T-cell Synthetic Outputs. Cancer Immunol Res 9, 1047–1060 (2021).3424429810.1158/2326-6066.CIR-20-0470PMC8415133

[56] SommermeyerD. Fully human CD19-specific chimeric antigen receptors for T-cell therapy. Leukemia 31, 2191–2199 (2017).2820295310.1038/leu.2017.57PMC5608623

[57] LiuZ. Systematic comparison of 2A peptides for cloning multi-genes in a polycistronic vector. Sci Rep 7, 2193 (2017).2852681910.1038/s41598-017-02460-2PMC5438344

[58] JonnalagaddaM. Efficient selection of genetically modified human T cells using methotrexate-resistant human dihydrofolate reductase. Gene Ther 20, 853–860 (2013).2330328210.1038/gt.2012.97PMC4028078

[59] WangX. A transgene-encoded cell surface polypeptide for selection, in vivo tracking, and ablation of engineered cells. Blood 118, 1255–1263 (2011).2165332010.1182/blood-2011-02-337360PMC3152493

