# Supplementary material for: Context-specific synthetic T cell promoters from assembled transcriptional elements
Source: Res Sq. 2023 Oct 17:rs.3.rs-3339290. Preprint. [Version 1] doi: 10.21203/rs.3.rs-3339290/v1 (PMC10602160; doi:10.21203/rs.3.rs-3339290/v1)
Supplement: Supplement 1 [file NIHPPRS3339290V1-supplement-1.pdf]

Supplementary Files

This is a list of supplementary files associated with this preprint. Click to download.

- SupplementaryTable1.csv
- SupplementaryTable2.xlsx
- SupplementaryTable3.xlsx
- SupplementaryTable4.xlsx
- SupplementalTable5Pvalues.xlsx
- SupplementalExtendedDataIndividualDonors.xlsx
- ExtendedDataFigures.docx
- SUPPLEMENTARYINFORMATION.docx
